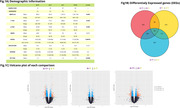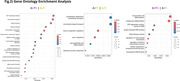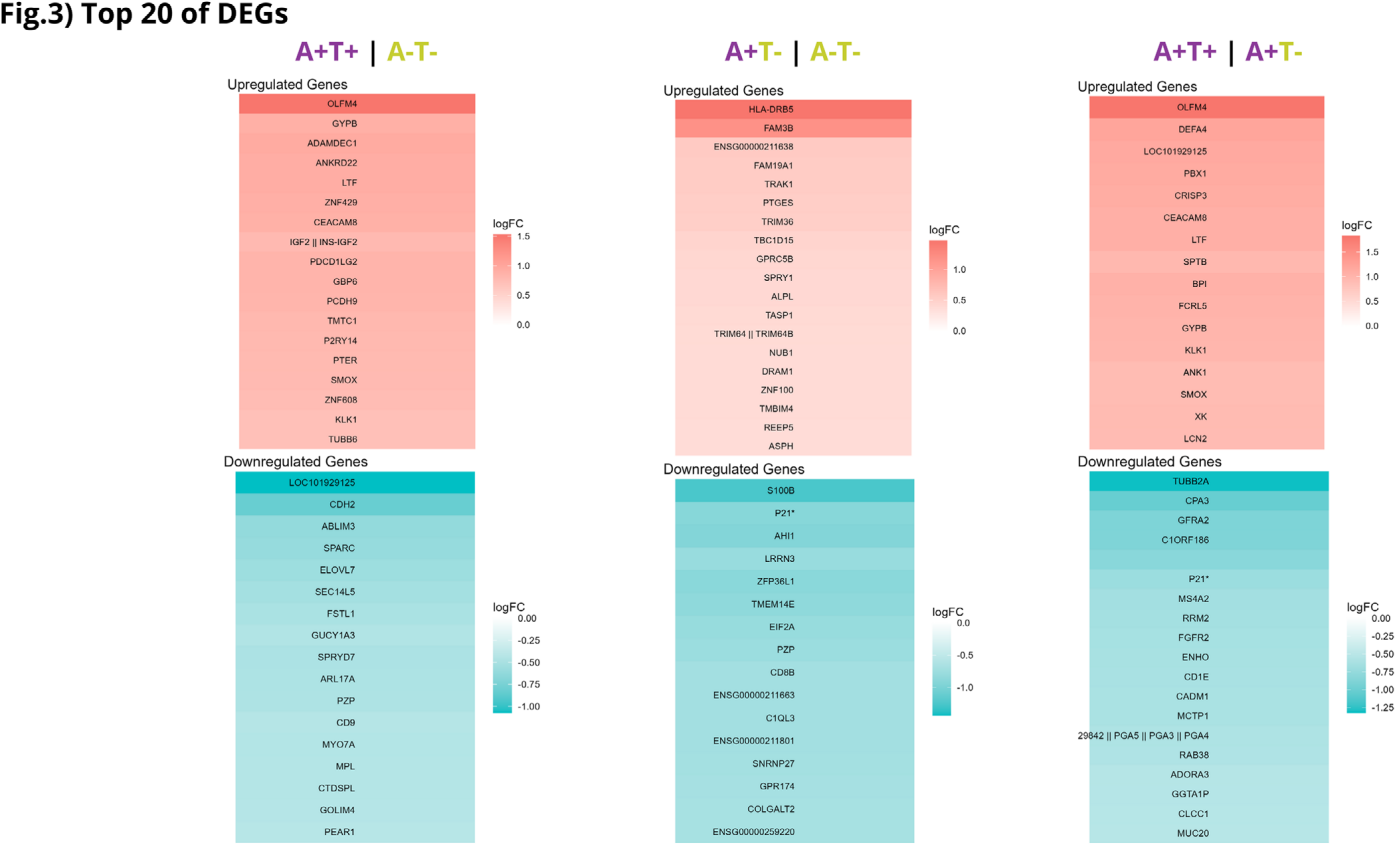# Blood transcriptomics of preclinical Alzheimer’s Disease patients

**DOI:** 10.1002/alz.095700

**Published:** 2025-01-09

**Authors:** Marcelo Madrid de Bittencourt, Marco De Bastiani, Pamela C.L. Ferreira, Eduardo R. Zimmer, João Batista Teixeira da Rocha

**Affiliations:** ^1^ Universidade Federal do Rio Grande do Sul (UFRGS), Porto Alegre, Rio Grande do Sul Brazil; ^2^ Universidade Federal do Rio Grande do Sul, Porto Alegre, Rio Grande do Sul Brazil; ^3^ University of Pittsburgh, Pittsburgh, PA USA; ^4^ Federal University of Rio Grande do Sul (UFRGS), Porto Alegre, RS Brazil

## Abstract

**Background:**

Blood transcriptomic differences have been described in patients with Alzheimer’s disease (AD). However, blood transcriptomic core molecular programs in cognitively unimpaired (CU) individuals positive to biomarkers of Amyloid and Tau pathology, defined as preclinical AD, remains to be explored. Therefore, we aimed to establish blood molecular core programs in preclinical AD.

**Method:**

We selected 63 CU individuals negative or positive to amyloid‐B (A) and Tau (T) but negative for neurodegeneration from the ADNI database, with blood transcriptomics available (Fig. 1A). We performed microarray data analysis in R, utilizing the *limma* method, for identifying differentially expressed genes (DEGs) in CU A‐T‐; CU A+T‐ and CU A+T+. Amyloid‐B and Tau positivity was defined based on CSF levels of Aß(1‐42) and p‐tau (cutoffs of 977 pg/mL for Aß and 24 pg/mL for Tau). Gene Ontology (GO) enrichment analysis was conducted for all categories, separately for each comparison. P‐value < 0.05 was considered statistically significant.

**Result:**

The comparison with the CU A‐T‐ group revealed 765 DEGs for CU A+T‐ and 1315 DEGs for CU A+T+. By comparing CU A+T+ with CU A+T‐, we identified 1171 DEGs. Only 29 DEGs were shared across all comparisons (Fig. 1B). Enrichment analysis identified 6 terms for CU A+T‐ versus CU A‐T‐, 17 terms for CU A+T+ versus CU A‐T‐, and 9 for CU A+T+ versus CU A+T‐. Only the second and the third comparisons shared the same terms, of which are “ATP‐dependent activity, acting on DNA“ and “single‐stranded DNA binding “. Among the non‐shared terms, CU A+T+ versus CU A‐T‐ included “ATP hydrolysis activity“, “ chromosomal region“, “tubulin binding“; for the CU A+T‐ versus CU A‐T‐, “cytoskeleton‐dependent intracellular transport“, “microtubule‐based transport“, “neuroprojection cytoplasm“; and for CU A+T+ versus CU A+T‐, “mononuclear cell differentiation”, “lymphocyte differentiation”, “regulation of macrophage chemotaxis”(Fig. 2).

**Conclusion:**

Our results suggest changes in blood transcriptomics among preclinical individuals with Amyloid and/or Tau pathology. Amyloid was related to disruptions in immune‐mediated and cytoskeleton proteins, while individuals positive for amyloid and Tau presented disturbances in inflammatory responses. Altogether, both Amyloid and Tau contribute to deregulations in cell cycle and DNA repair systems compared with CU individuals with no pathology.